# 2-Benzyl­sulfanyl-3-(2,2,2-trifluoro­ethoxy)pyridine

**DOI:** 10.1107/S1600536810043308

**Published:** 2010-11-06

**Authors:** Zhi-Qiang Feng, Xiao-Li Yang, Yuan-Feng Ye, Tao Dong

**Affiliations:** a College of Materials Engineering, Jinling Institute of Technology, No. 99 Hongjing Street, Nanjing, Nanjing 211169, People’s Republic of China

## Abstract

The title compound, C_14_H_12_F_3_NOS, was synthesized by the reaction of 2-chloro-3-(2,2,2-trifluoro­eth­oxy)pyridine and phenyl­methane­thiol. The dihedral angle between the aromatic rings is 76.7 (2)°. In the crystal structure, weak aromatic π–π stacking between inversion-related pairs of pyridine rings [centroid-to-centroid separation = 3.776 (2) Å] may help to establish the packing.

## Related literature

For background to the title compound as a precursor of weedkillers, see: Howard *et al.* (2001 ▶). For reference bond-length data, see: Allen *et al.* (1987 ▶).
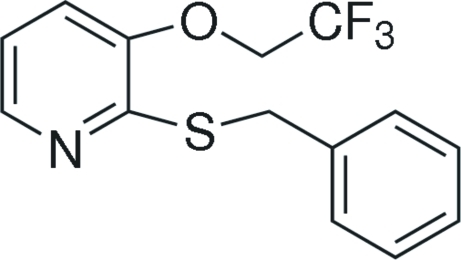


## Experimental

### 

#### Crystal data

C_14_H_12_F_3_NOS
*M* *_r_* = 299.31
Monoclinic, 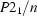

*a* = 8.3770 (17) Å
*b* = 16.860 (3) Å
*c* = 10.144 (2) Å
β = 97.32 (3)°
*V* = 1421.0 (5) Å^3^
*Z* = 4
Mo *K*α radiation
μ = 0.26 mm^−1^
*T* = 293 K
0.30 × 0.20 × 0.20 mm

#### Data collection

Enraf–Nonius CAD-4 diffractometer
Absorption correction: ψ scan (North *et al.*, 1968 ▶) *T*_min_ = 0.927, *T*_max_ = 0.951
2758 measured reflections
2575 independent reflections
1716 reflections with *I* > 2σ(*I*)
*R*_int_ = 0.036
3 standard reflections every 200 reflections  intensity decay: none

#### Refinement

*R*[*F* ^2^ > 2σ(*F* ^2^)] = 0.058
*wR*(*F* ^2^) = 0.136
*S* = 1.01
2575 reflections
182 parameters
H-atom parameters constrained
Δρ_max_ = 0.26 e Å^−3^
Δρ_min_ = −0.28 e Å^−3^

### 

Data collection: *CAD-4 Software* (Enraf–Nonius, 1989 ▶); cell refinement: *CAD-4 Software*; data reduction: *XCAD4* (Harms & Wocadlo, 1995 ▶); program(s) used to solve structure: *SHELXTL* (Sheldrick, 2008 ▶); program(s) used to refine structure: *SHELXTL*; molecular graphics: *SHELXTL*; software used to prepare material for publication: *SHELXTL*.

## Supplementary Material

Crystal structure: contains datablocks global, I. DOI: 10.1107/S1600536810043308/hb5702sup1.cif

Structure factors: contains datablocks I. DOI: 10.1107/S1600536810043308/hb5702Isup2.hkl

Additional supplementary materials:  crystallographic information; 3D view; checkCIF report

## Figures and Tables

**Fig. 1.**
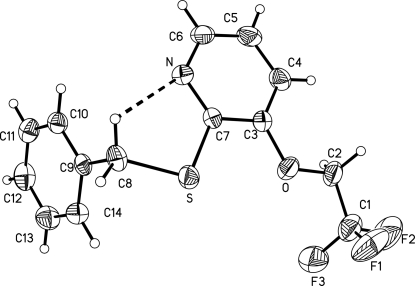
The molecular structure of the title molecule, with displacement ellipsoids drawn at the 50% probability level.

## supplementary crystallographic information

### Comment

3-(2,2,2-trifluoroethoxy)-2-(benzylthio)pyridine, (I), is an important intermediate for a novel weed killer N-[[(4,6-Dimethoxy-2-Pyrimidinyl)Amino]Carbonyl]- 3-(2,2,2-Trifluoroethoxy)-2-Pyridinesulfonamide, which is of high herbicidal activity and friendly to the environment (Howard *et al.*, 2001).

Here we report the crystal structure of (I). In the molecule of the title compound (Fig. 1), all bond lengths and angles (Allen *et al.*, 1987) are within normal ranges. Rings A (C3–C6/N/C7) and B (C9–C14) are of course planar, while the dihedral angle between them is 76.7 (2)°.

### Experimental

3-(2,2,2-trifluoroethoxy)-2-chloropyridine (10 mmol) and potassium carbonate (20 mmol) were dissolved in DMF (20 ml), and the mixture was stirred at reflux for 1 h. Then phenylmethanethiol (12 mmol) was added dropwise to the mixture above, refluxed for another 6 h. After cooling and filtering, crude compound (I) was obtained. Pure product suitable for X-ray diffraction was recrystallized from alcohol as colourless blocks of (I).

### Refinement

All H atoms bonded to the C atoms were placed geometrically at the distances of 0.93–0.97 Å and included in the refinement in riding motion approximation with *U*_iso_(H) = 1.2 *U*_eq_ of the carrier atom.

### Crystal data

**Table:**

C_14_H_12_F_3_NOS	*F*(000) = 616
*M**_r_* = 299.31	*D*_x_ = 1.399 Mg m^−^^3^
Monoclinic, *P*2_1_/*n*	Melting point: 353 K
Hall symbol: -P 2yn	Mo *K*α radiation, λ = 0.71073 Å
*a* = 8.3770 (17) Å	Cell parameters from 25 reflections
*b* = 16.860 (3) Å	θ = 9–13°
*c* = 10.144 (2) Å	µ = 0.26 mm^−^^1^
β = 97.32 (3)°	*T* = 293 K
*V* = 1421.0 (5) Å^3^	Block, colourless
*Z* = 4	0.30 × 0.20 × 0.20 mm

### Data collection

**Table:**

Enraf–Nonius CAD-4 diffractometer	1716 reflections with *I* > 2σ(*I*)
Radiation source: fine-focus sealed tube	*R*_int_ = 0.036
graphite	θ_max_ = 25.3°, θ_min_ = 2.4°
ω/2θ scans	*h* = 0→10
Absorption correction: ψ scan (North *et al.*, 1968)	*k* = 0→20
*T*_min_ = 0.927, *T*_max_ = 0.951	*l* = −12→12
2758 measured reflections	3 standard reflections every 200 reflections
2575 independent reflections	intensity decay: none

### Refinement

**Table:**

Refinement on *F*^2^	Secondary atom site location: difference Fourier map
Least-squares matrix: full	Hydrogen site location: inferred from neighbouring sites
*R*[*F*^2^ > 2σ(*F*^2^)] = 0.058	H-atom parameters constrained
*wR*(*F*^2^) = 0.136	*w* = 1/[σ^2^(*F*_o_^2^) + (0.027*P*)^2^ + 2.174*P*] where *P* = (*F*_o_^2^ + 2*F*_c_^2^)/3
*S* = 1.01	(Δ/σ)_max_ < 0.001
2575 reflections	Δρ_max_ = 0.26 e Å^−^^3^
182 parameters	Δρ_min_ = −0.28 e Å^−^^3^
0 restraints	Extinction correction: *SHELXTL* (Sheldrick, 2008), Fc^*^=kFc[1+0.001xFc^2^λ^3^/sin(2θ)]^-1/4^
Primary atom site location: structure-invariant direct methods	Extinction coefficient: 0.049 (2)

### Special details

**Table:**

Geometry. All esds (except the esd in the dihedral angle between two l.s. planes) are estimated using the full covariance matrix. The cell esds are taken into account individually in the estimation of esds in distances, angles and torsion angles; correlations between esds in cell parameters are only used when they are defined by crystal symmetry. An approximate (isotropic) treatment of cell esds is used for estimating esds involving l.s. planes.
Refinement. Refinement of F^2^ against ALL reflections. The weighted R-factor wR and goodness of fit S are based on F^2^, conventional R-factors R are based on F, with F set to zero for negative F^2^. The threshold expression of F^2^ > 2sigma(F^2^) is used only for calculating R-factors(gt) etc. and is not relevant to the choice of reflections for refinement. R-factors based on F^2^ are statistically about twice as large as those based on F, and R- factors based on ALL data will be even larger.

### Fractional atomic coordinates and isotropic or equivalent isotropic displacement parameters (Å^2^)

**Table:**

	*x*	*y*	*z*	*U*_iso_*/*U*_eq_	
S1	0.18955 (13)	0.37494 (6)	0.26098 (10)	0.0562 (3)	
O1	−0.0642 (3)	0.33867 (16)	0.0614 (3)	0.0602 (8)	
N1	0.2971 (4)	0.45168 (18)	0.0576 (3)	0.0516 (8)	
F1	−0.2695 (4)	0.21214 (19)	0.1032 (4)	0.1275 (14)	
F2	−0.4629 (4)	0.2729 (2)	−0.0088 (4)	0.1367 (14)	
F3	−0.3439 (4)	0.3240 (2)	0.1674 (3)	0.1163 (11)	
C1	−0.3180 (6)	0.2823 (3)	0.0625 (6)	0.0831 (15)	
C2	−0.2097 (5)	0.3222 (3)	−0.0197 (4)	0.0667 (12)	
H2A	−0.1897	0.2882	−0.0930	0.080*	
H2B	−0.2582	0.3710	−0.0560	0.080*	
C3	0.0459 (4)	0.3844 (2)	0.0070 (4)	0.0463 (9)	
C4	0.0345 (5)	0.4081 (3)	−0.1235 (4)	0.0607 (11)	
H4A	−0.0530	0.3936	−0.1846	0.073*	
C5	0.1580 (5)	0.4542 (3)	−0.1607 (4)	0.0678 (12)	
H5A	0.1544	0.4714	−0.2482	0.081*	
C6	0.2844 (5)	0.4742 (2)	−0.0694 (4)	0.0592 (11)	
H6A	0.3661	0.5051	−0.0966	0.071*	
C7	0.1804 (4)	0.4077 (2)	0.0952 (3)	0.0427 (8)	
C8	0.3643 (5)	0.4284 (2)	0.3398 (4)	0.0550 (10)	
H8A	0.4075	0.4004	0.4201	0.066*	
H8B	0.4463	0.4285	0.2804	0.066*	
C9	0.3294 (4)	0.5127 (2)	0.3752 (3)	0.0432 (9)	
C10	0.4057 (4)	0.5758 (2)	0.3243 (4)	0.0499 (9)	
H10A	0.4780	0.5663	0.2637	0.060*	
C11	0.3776 (5)	0.6527 (2)	0.3611 (4)	0.0591 (11)	
H11A	0.4310	0.6943	0.3254	0.071*	
C12	0.2721 (5)	0.6681 (3)	0.4494 (5)	0.0704 (13)	
H12A	0.2528	0.7200	0.4739	0.084*	
C13	0.1939 (5)	0.6058 (3)	0.5023 (5)	0.0730 (13)	
H13A	0.1226	0.6157	0.5635	0.088*	
C14	0.2214 (5)	0.5291 (3)	0.4647 (4)	0.0593 (11)	
H14A	0.1668	0.4877	0.4997	0.071*	

### Atomic displacement parameters (Å^2^)

**Table:**

	*U*^11^	*U*^22^	*U*^33^	*U*^12^	*U*^13^	*U*^23^
S1	0.0704 (7)	0.0507 (6)	0.0449 (5)	−0.0104 (5)	−0.0025 (5)	0.0047 (5)
O1	0.0537 (16)	0.0634 (17)	0.0608 (17)	−0.0163 (14)	−0.0029 (13)	0.0096 (14)
N1	0.0501 (19)	0.054 (2)	0.0519 (19)	−0.0038 (16)	0.0119 (15)	−0.0019 (16)
F1	0.113 (3)	0.073 (2)	0.206 (4)	−0.0068 (19)	0.059 (3)	0.037 (2)
F2	0.063 (2)	0.165 (4)	0.182 (4)	−0.039 (2)	0.013 (2)	−0.008 (3)
F3	0.119 (3)	0.121 (3)	0.120 (3)	−0.018 (2)	0.055 (2)	−0.021 (2)
C1	0.063 (3)	0.076 (4)	0.114 (4)	−0.014 (3)	0.024 (3)	−0.016 (3)
C2	0.054 (3)	0.065 (3)	0.080 (3)	−0.016 (2)	0.005 (2)	−0.012 (2)
C3	0.045 (2)	0.046 (2)	0.047 (2)	−0.0015 (17)	0.0027 (17)	−0.0018 (17)
C4	0.059 (3)	0.076 (3)	0.044 (2)	−0.002 (2)	−0.0020 (19)	−0.001 (2)
C5	0.077 (3)	0.084 (3)	0.043 (2)	−0.002 (3)	0.012 (2)	0.010 (2)
C6	0.060 (3)	0.065 (3)	0.056 (3)	−0.004 (2)	0.022 (2)	0.002 (2)
C7	0.044 (2)	0.0412 (19)	0.0415 (19)	0.0020 (16)	0.0023 (16)	−0.0037 (16)
C8	0.059 (2)	0.057 (2)	0.045 (2)	0.004 (2)	−0.0091 (18)	−0.0034 (18)
C9	0.0396 (19)	0.051 (2)	0.0363 (19)	0.0028 (17)	−0.0076 (15)	−0.0015 (16)
C10	0.044 (2)	0.058 (2)	0.045 (2)	0.0031 (19)	−0.0003 (17)	0.0016 (18)
C11	0.054 (2)	0.055 (2)	0.065 (3)	0.000 (2)	−0.006 (2)	0.008 (2)
C12	0.067 (3)	0.057 (3)	0.083 (3)	0.011 (2)	−0.005 (3)	−0.015 (2)
C13	0.067 (3)	0.083 (3)	0.072 (3)	0.007 (3)	0.019 (2)	−0.017 (3)
C14	0.059 (3)	0.064 (3)	0.056 (2)	−0.004 (2)	0.011 (2)	0.001 (2)

### Geometric parameters (Å, °)

**Table:**

S1—C7	1.762 (4)	C5—H5A	0.9300
S1—C8	1.816 (4)	C6—H6A	0.9300
O1—C3	1.371 (4)	C8—C9	1.504 (5)
O1—C2	1.409 (4)	C8—H8A	0.9700
N1—C7	1.321 (4)	C8—H8B	0.9700
N1—C6	1.334 (5)	C9—C10	1.375 (5)
F1—C1	1.300 (6)	C9—C14	1.389 (5)
F2—C1	1.341 (6)	C10—C11	1.378 (5)
F3—C1	1.316 (6)	C10—H10A	0.9300
C1—C2	1.472 (6)	C11—C12	1.361 (6)
C2—H2A	0.9700	C11—H11A	0.9300
C2—H2B	0.9700	C12—C13	1.382 (6)
C3—C4	1.374 (5)	C12—H12A	0.9300
C3—C7	1.403 (5)	C13—C14	1.375 (6)
C4—C5	1.385 (6)	C13—H13A	0.9300
C4—H4A	0.9300	C14—H14A	0.9300
C5—C6	1.358 (5)		
			
C7—S1—C8	101.50 (18)	N1—C7—C3	122.4 (3)
C3—O1—C2	116.9 (3)	N1—C7—S1	120.5 (3)
C7—N1—C6	117.9 (3)	C3—C7—S1	117.0 (3)
F1—C1—F3	107.8 (5)	C9—C8—S1	113.8 (3)
F1—C1—F2	106.8 (4)	C9—C8—H8A	108.8
F3—C1—F2	105.6 (4)	S1—C8—H8A	108.8
F1—C1—C2	113.9 (4)	C9—C8—H8B	108.8
F3—C1—C2	113.0 (4)	S1—C8—H8B	108.8
F2—C1—C2	109.2 (5)	H8A—C8—H8B	107.7
O1—C2—C1	108.0 (4)	C10—C9—C14	117.7 (4)
O1—C2—H2A	110.1	C10—C9—C8	121.8 (3)
C1—C2—H2A	110.1	C14—C9—C8	120.5 (4)
O1—C2—H2B	110.1	C9—C10—C11	121.5 (4)
C1—C2—H2B	110.1	C9—C10—H10A	119.2
H2A—C2—H2B	108.4	C11—C10—H10A	119.2
O1—C3—C4	125.7 (3)	C12—C11—C10	120.3 (4)
O1—C3—C7	115.3 (3)	C12—C11—H11A	119.8
C4—C3—C7	119.0 (3)	C10—C11—H11A	119.8
C3—C4—C5	117.6 (4)	C11—C12—C13	119.4 (4)
C3—C4—H4A	121.2	C11—C12—H12A	120.3
C5—C4—H4A	121.2	C13—C12—H12A	120.3
C6—C5—C4	119.9 (4)	C14—C13—C12	120.2 (4)
C6—C5—H5A	120.1	C14—C13—H13A	119.9
C4—C5—H5A	120.1	C12—C13—H13A	119.9
N1—C6—C5	123.2 (4)	C13—C14—C9	121.0 (4)
N1—C6—H6A	118.4	C13—C14—H14A	119.5
C5—C6—H6A	118.4	C9—C14—H14A	119.5
			
C3—O1—C2—C1	−172.3 (4)	O1—C3—C7—S1	0.3 (4)
F1—C1—C2—O1	−66.8 (6)	C4—C3—C7—S1	−179.5 (3)
F3—C1—C2—O1	56.8 (6)	C8—S1—C7—N1	7.5 (3)
F2—C1—C2—O1	174.0 (4)	C8—S1—C7—C3	−173.0 (3)
C2—O1—C3—C4	−7.7 (6)	C7—S1—C8—C9	81.3 (3)
C2—O1—C3—C7	172.6 (3)	S1—C8—C9—C10	−120.4 (3)
O1—C3—C4—C5	−179.8 (4)	S1—C8—C9—C14	61.7 (4)
C7—C3—C4—C5	−0.1 (6)	C14—C9—C10—C11	0.5 (5)
C3—C4—C5—C6	0.1 (6)	C8—C9—C10—C11	−177.5 (3)
C7—N1—C6—C5	0.1 (6)	C9—C10—C11—C12	−0.1 (6)
C4—C5—C6—N1	−0.1 (7)	C10—C11—C12—C13	0.3 (6)
C6—N1—C7—C3	−0.1 (5)	C11—C12—C13—C14	−0.8 (7)
C6—N1—C7—S1	179.5 (3)	C12—C13—C14—C9	1.1 (7)
O1—C3—C7—N1	179.8 (3)	C10—C9—C14—C13	−0.9 (6)
C4—C3—C7—N1	0.1 (6)	C8—C9—C14—C13	177.1 (4)
